# Supplementary material for: Lysosome-Disrupting Agents in Combination with Venetoclax Increase Apoptotic Response in Primary Chronic Lymphocytic Leukemia (CLL) Cells Mediated by Lysosomal Cathepsin D Release and Inhibition of Autophagy
Source: Cells. 2024 Jun 15;13(12):1041. doi: 10.3390/cells13121041 (PMC11202145; doi:10.3390/cells13121041)
Supplement: Supplementary file 1 [file cells-13-01041-s001.zip › cells-2946598-supplementary.pdf]

Supplementary table S1

Supplementary table S1: Clinical data on CLL patients' sample used. ND- Not determined

Study ID	Sex	Age	Past Treatment for CLL	IGHV	RAI stage	FISH Status
EB052CLL	Male	72	NO	Unmutated	4	11q22 del, 13q14 del
EB053CLL	Male	73	NO	ND	0	13q14 del
EB054CLL	Male	63	YES	ND	4	13q14 del, 17p13 del
EB055CLL	Male	65	YES	Unmutated	4	Trisomy 12
EB056CLL	Male	74	NO	Mutated	1	Trisomy 12
EB057CLL	Male	54	NO	Unmutated	1	Trisomy 12
EB060CLL	Male	56	NO	ND	1	ND
EB062CLL	Female	82	NO	ND	0	ND
EB063CLL	Male	78	NO	ND	1	ND
EB064CLL	Male	50	NO	ND	1	ND
EB065CLL	Male	68	NO	Unmutated	4	Normal
EB066CLL	Female	48	NO	ND	0	ND
EB069CLL	Male	81	NO	Unmutated	3	Normal
EB070CLL	Male	65	NO	Unmutated	4	Normal
EB071CLL	Male	62	NO	ND	2	ND
EB072CLL	Male	39	NO	ND	1	Normal
EB074CLL	Male	54	NO	ND	1	13q14 del
EB075CLL	Female	64	NO	Mutated	1	13q14 del
EB076CLL	Female	77	NO	ND	1	ND
EB078CLL	Female	84	NO	Unmutated	3	13q14 del
EB079CLL	Male	47	NO	Mutated	2	ND
EB080CLL	Female	73	NO	Unmutated	1	Trisomy 12
EB081CLL	Male	61	NO	Mutated	2	Normal
EB082CLL	Female	71	NO	ND	0	ND
EB083CLL	Female	85	NO	ND	0	ND
EB084CLL	Female	77	NO	ND	1	ND
EB085CLL	Male	89	YES	ND	1	ND
EB086CLL	Male	71	NO	Mutated	0	13q14 del
EB087CLL	Female	68	YES	ND	1	17p13 del
EB088CLL	Female	76	NO	ND	0	Normal
EB089CLL	Male	80	NO	ND	0	ND
EB090CLL	Male	61	NO	ND	2	ND
EB091CLL	Male	58	NO	Mutated	0	13q14 del
EB092CLL	Male	64	NO	ND	1	ND
EB093CLL	Female	67	NO	ND	1	ND
EB094CLL	Male	70	NO	ND	0	ND
EB096CLL	Male	64	NO	ND	1	ND
EB097CLL	Male	77	NO	Mutated	3	17p13 del
EB098CLL	Male	61	NO	ND	2	Normal
EB099CLL	Male	85	NO	Unmutated	2	Normal
EB100CLL	Female	73	NO	ND	1	ND
EB101CLL	Female	72	NO	ND	0	ND

Supplementary Figure S1

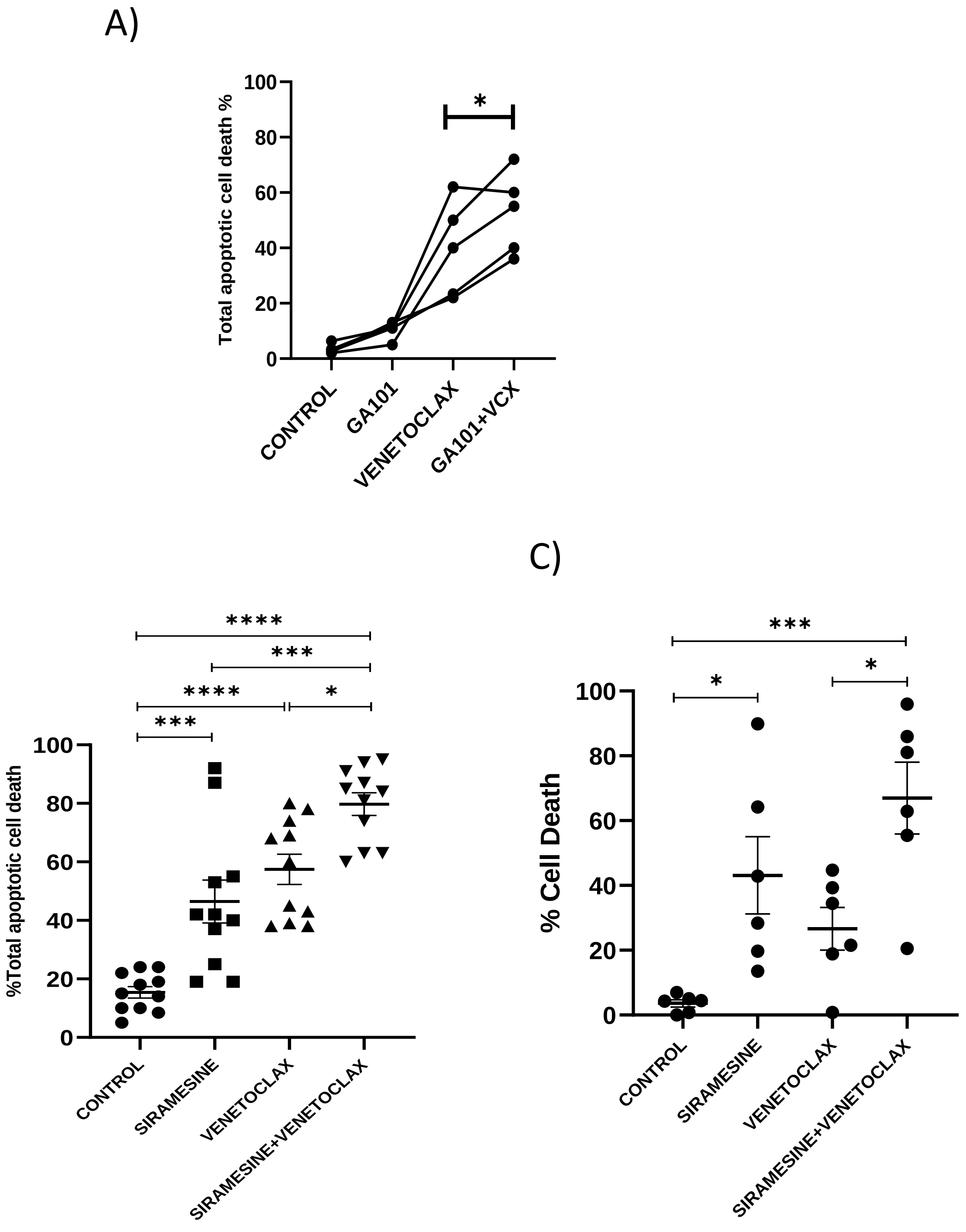

Supplementary figure S1: Siramesine or GA101 in combination with Venetoclax induces apoptosis in CLL cells. A) Primary CLL cells were treated with 50µg GA101 and 0.05nM venetoclax. Annexin V/7AAD assay was performed. N=5 independent different CLL patients denoted by line were analyzed. B) Primary CLL cells were treated with 1µM siramesine, 0.05nM venetoclax alone and in combination for 24 hours. Cells were stained with AnnexinV/7-AAD and fluorescence was measured using flow cytometry (N=11 independent different patient samples). C) CLL cells were treated with 1µM of siramesine and 0.05nM of venetoclax for 48 hours and, trypan blue exclusion assay was performed (N=6 independent different patient samples). Error bars represent SEM

Supplementary Figure S2

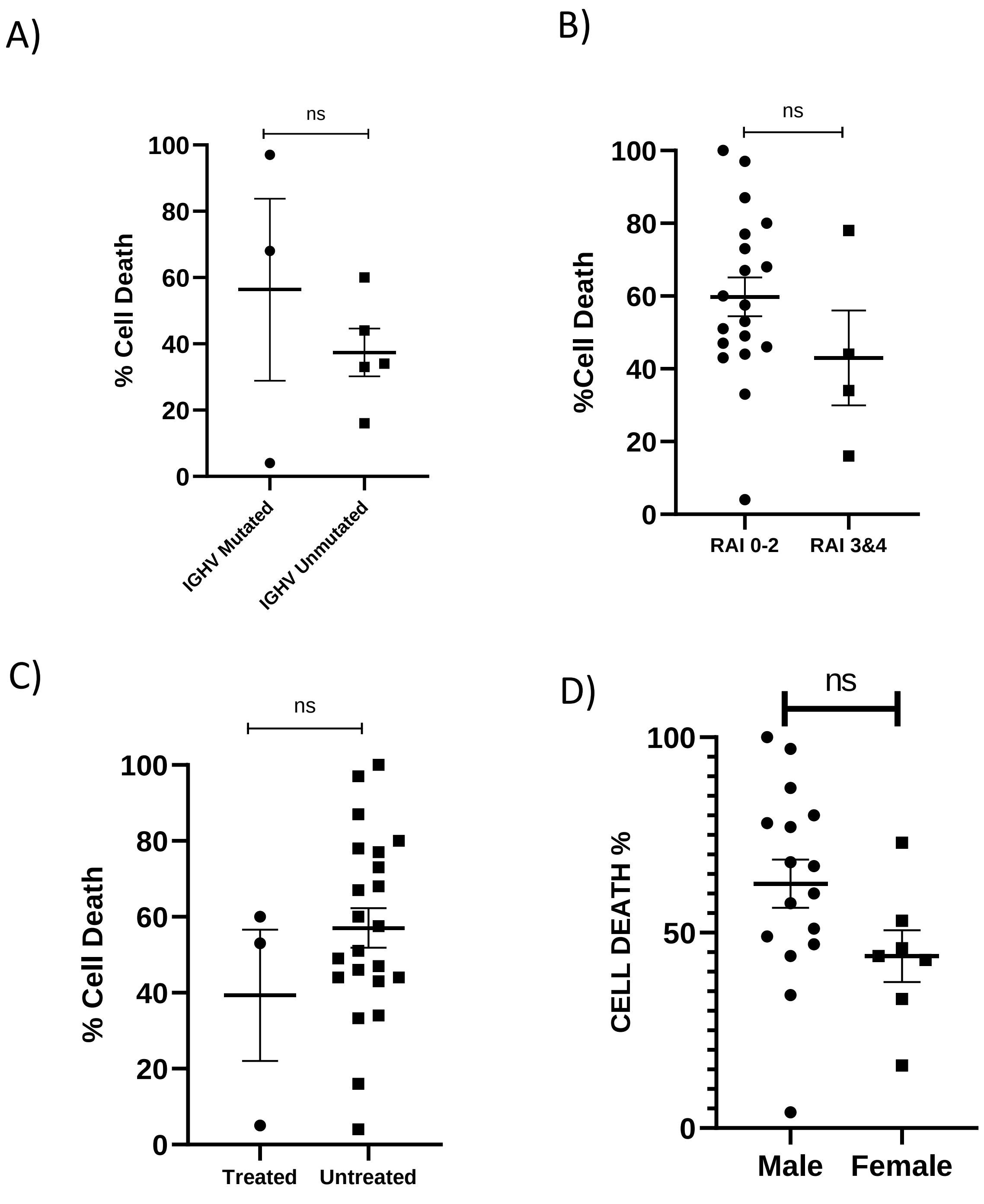

Supplementary figure S2: Characterization of patients based on various clinical classifications. Percentage of cell death based on trypan blue exclusion assay for combination of 1µM siramesine and 0.05nM venetoclax was performed for 24 hours A) The graph represents the classification of patients based on IGHV mutated or unmutated on the x-axis. B) The graph represents the classification of patients based on Rai stage 0-2 and Rai stage 3&4 on x-axis. C) The graph represents the classification of patients based on past history of treatment or not on x-axis. D) The graph represents whether CLL patients were male or female. Error bars represent SEM.

Supplementary Figure S3

A)

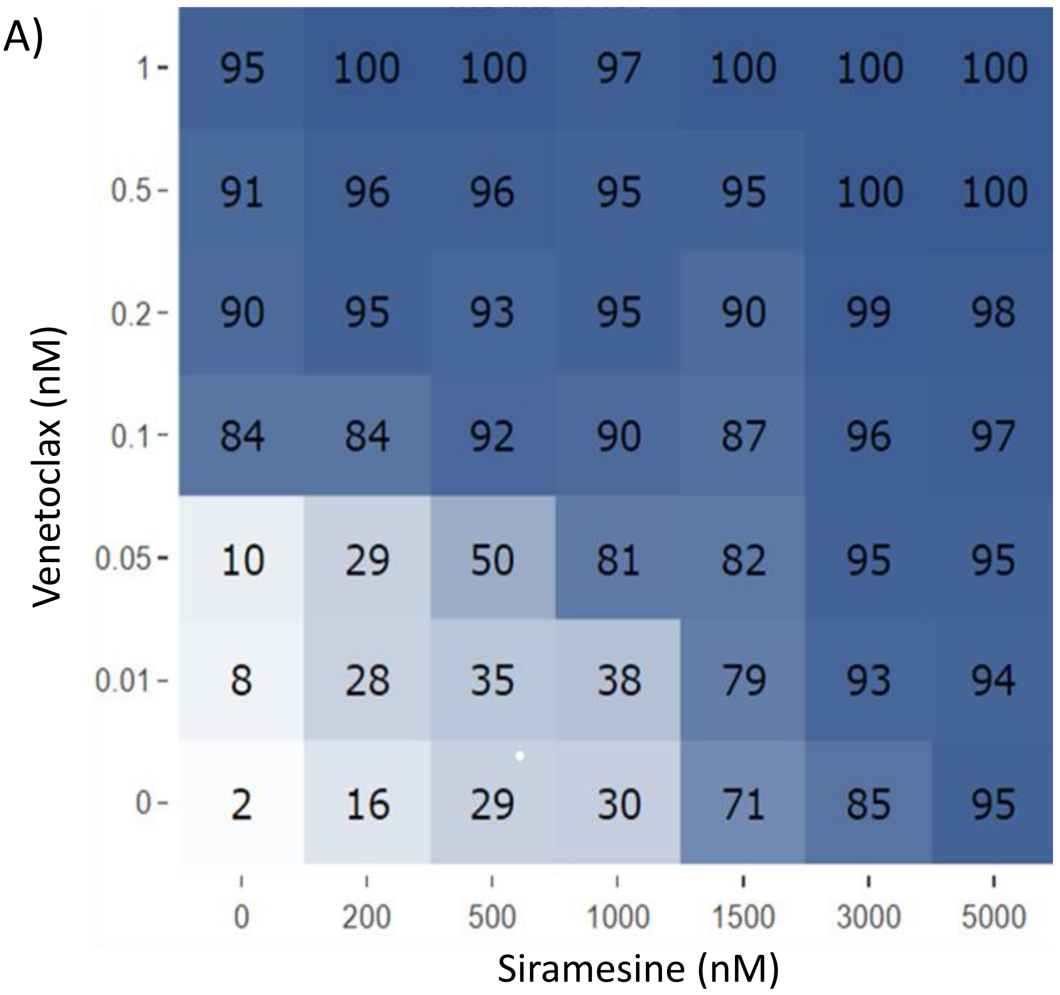

B)

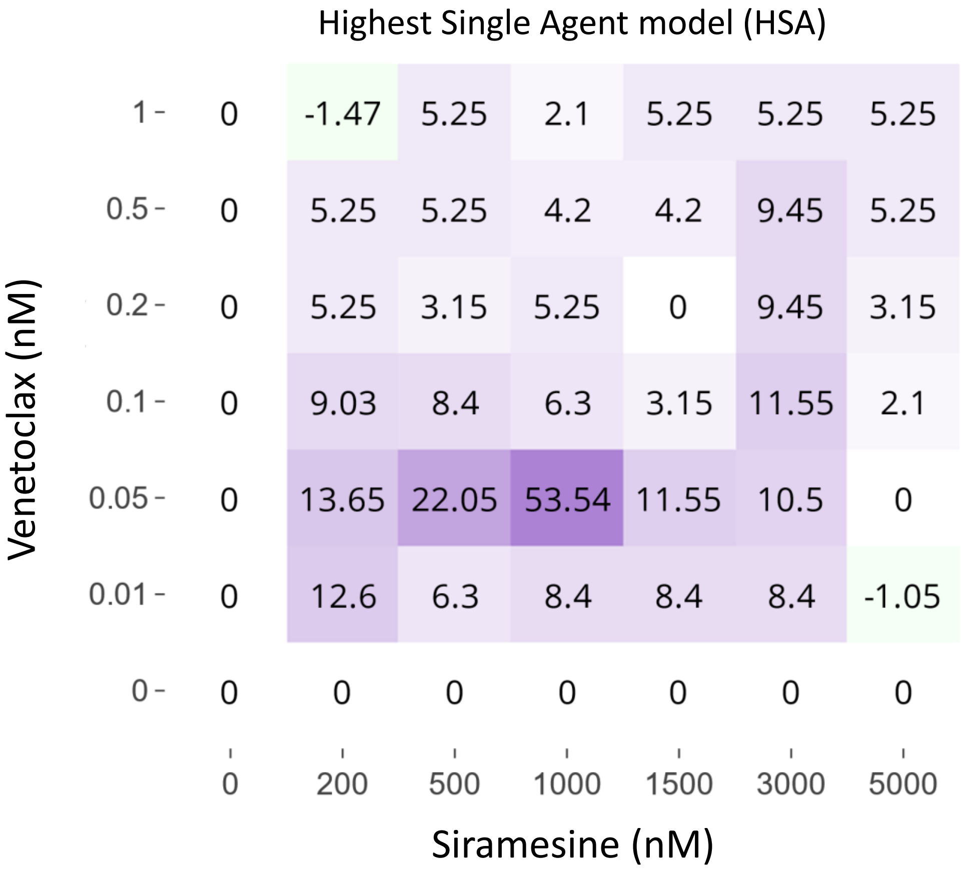

Supplementary figure S3: Siramesine gives synergistic cell death response when combined with Venetoclax. Percentage of cell death based on trypan blue exclusion assay for combination of siramesine and venetoclax with various doses alone and in combination was performed for 24 hours. Representative example of A) SynergyFinder+ dose response matrix was generated based on the individual dose response matrix. B) Highest single agent model was generated using Synergyfinder+ platform. N=3 [illegible]

Supplementary Figure S4

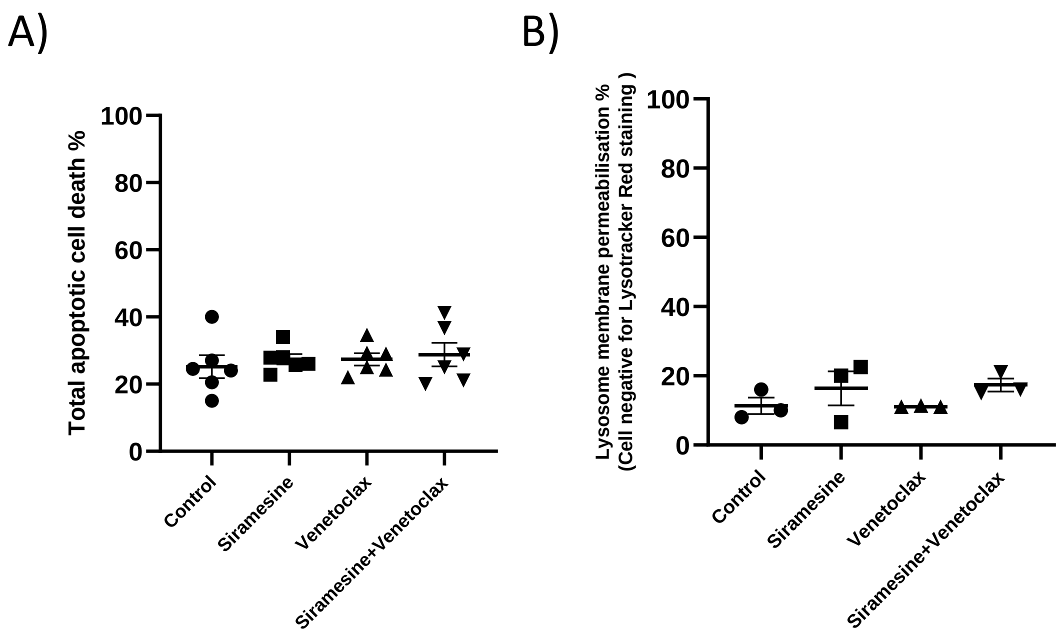

Supplementary figure S4: Siramesine in combination with Venetoclax fails to induce apoptosis in normal B cells. A) Normal B cells isolated from healthy volunteers were treated with 1µM siramesine and 0.05nM venetoclax. Annexin V/7AAD assay was performed. N=6 independent different healthy donor samples. B) The cells were treated with 1µM siramesine, 0.05nM venetoclax alone and in combination for 24 hours. Cells were stained with Lysotracker stain and fluorescence was measured using flow cytometry (N=6 independent different patient samples).

Supplementary Figure S5

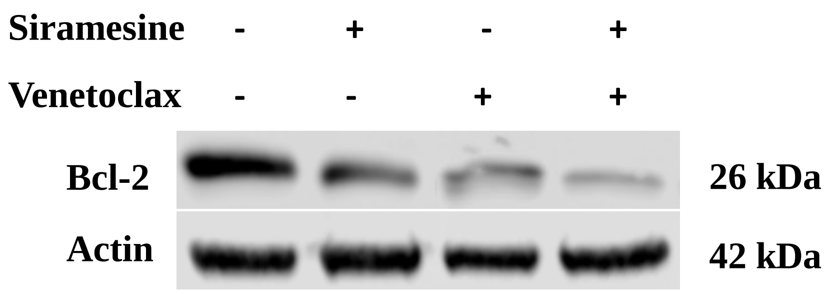

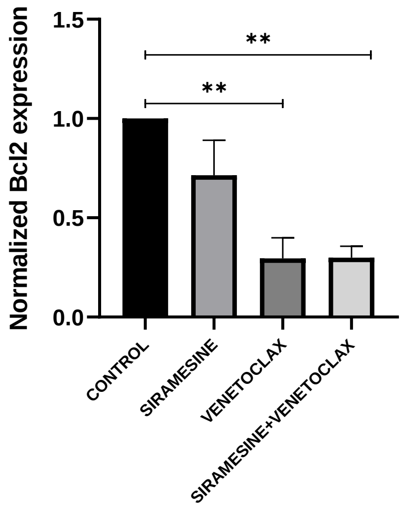

Supplementary figure S5: Inhibition of Bcl-2 by the combination of siramesine and venetoclax. CLL cells treated with 1μM siramesine, 0.05nM venetoclax alone and in combination for 24 hours. After treatment, cells were lysed, western blot determination of Bcl-2 was performed and actin was used as loading control. Densitometry was calculated (n=3).

Supplementary Figure S6

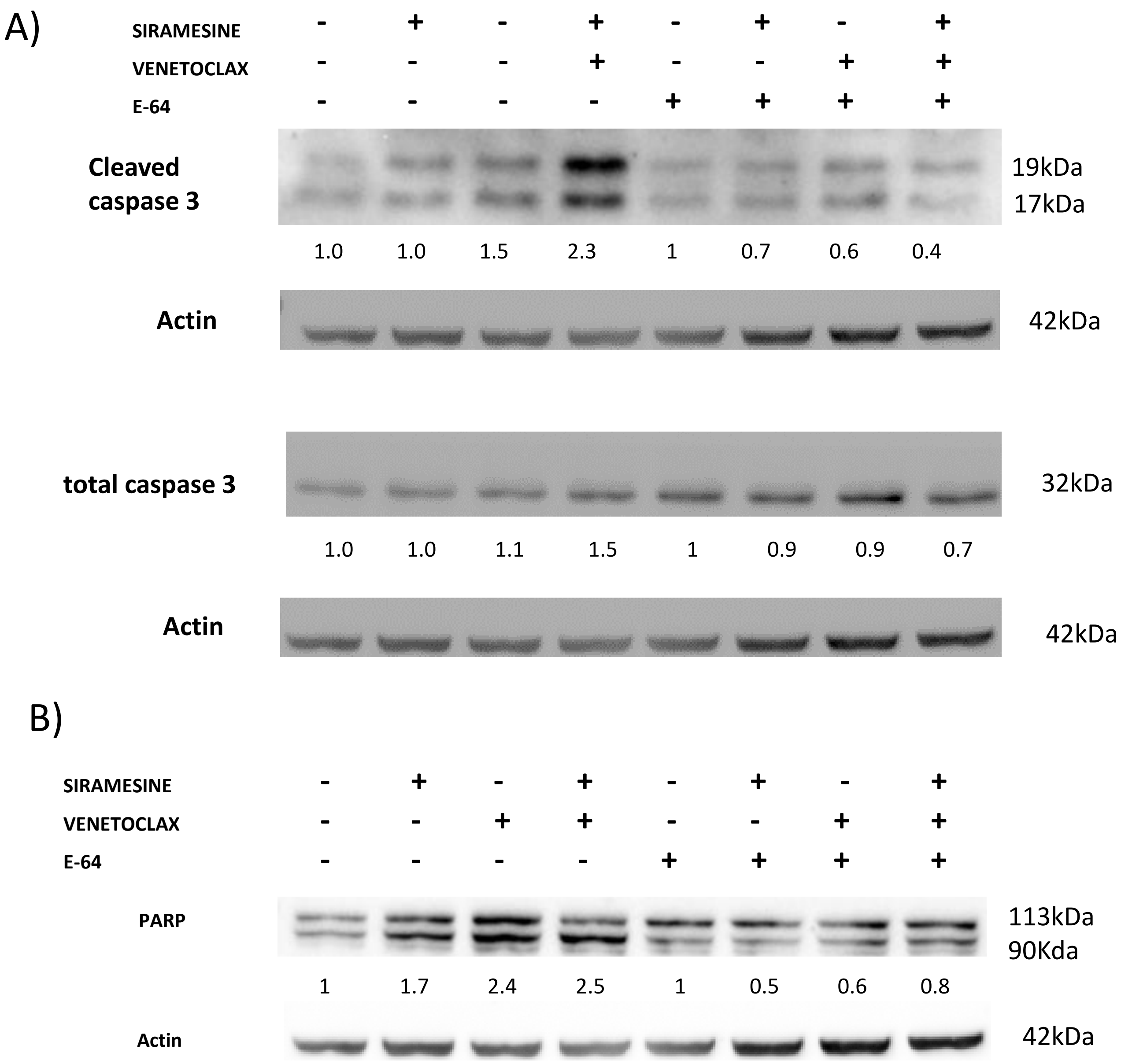

Supplementary figure S6: Cathepsin inhibitor E-64 blocks caspase 3 and PARP cleavage following treatment with siramesine and venetoclax. CLL cells treated with 1µM siramesine, 0.05nM venetoclax alone and in combination in the presence or absence of cathepsin inhibitor E-64 for 6 hours. A) After treatment, cells were lysed, western blot determination of total caspase 3 and cleaved caspase 3 using specific antibodies and actin was used as loading control. B) Western blot for PARP was performed on the lysate mentioned above and actin was used a loading control. Densitometry relative values are shown below.

Supplementary Figure S7

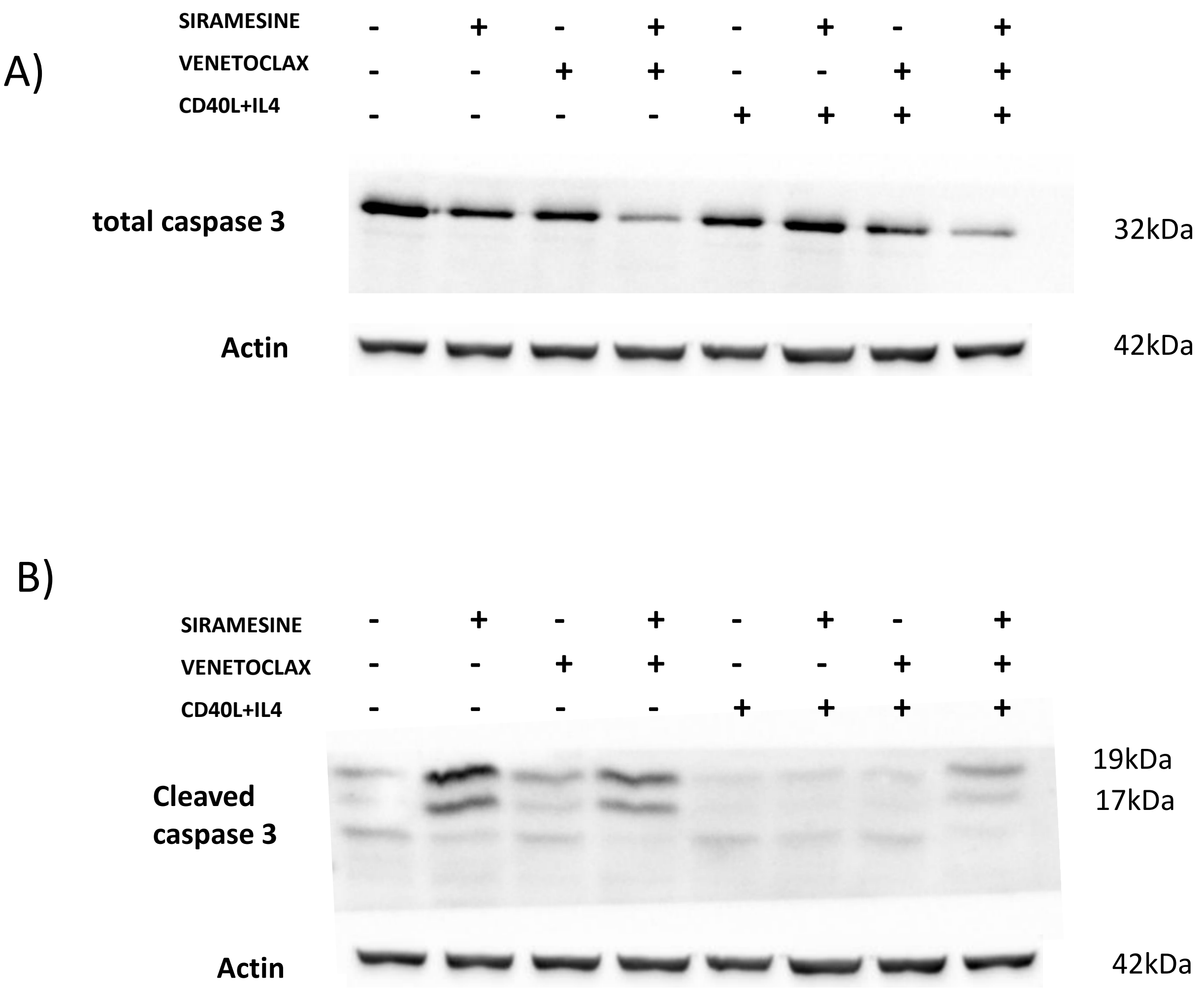

Supplementary figure S7: Treatment with IL4 and CD40L failed to block caspase 3 cleavage following treatment with siramesine and venetoclax. CLL cells treated with 1µM siramesine, 0.05nM venetoclax alone and in combination in the presence or absence of CD40L + IL4 for 24 hours. A) After treatment, cells were lysed, western blot determination of A) total caspase 3 and B) cleaved caspase 3 using specific antibodies. Actin was used as loading control. Densitometry relative values are shown below.

Supplementary Figure S8

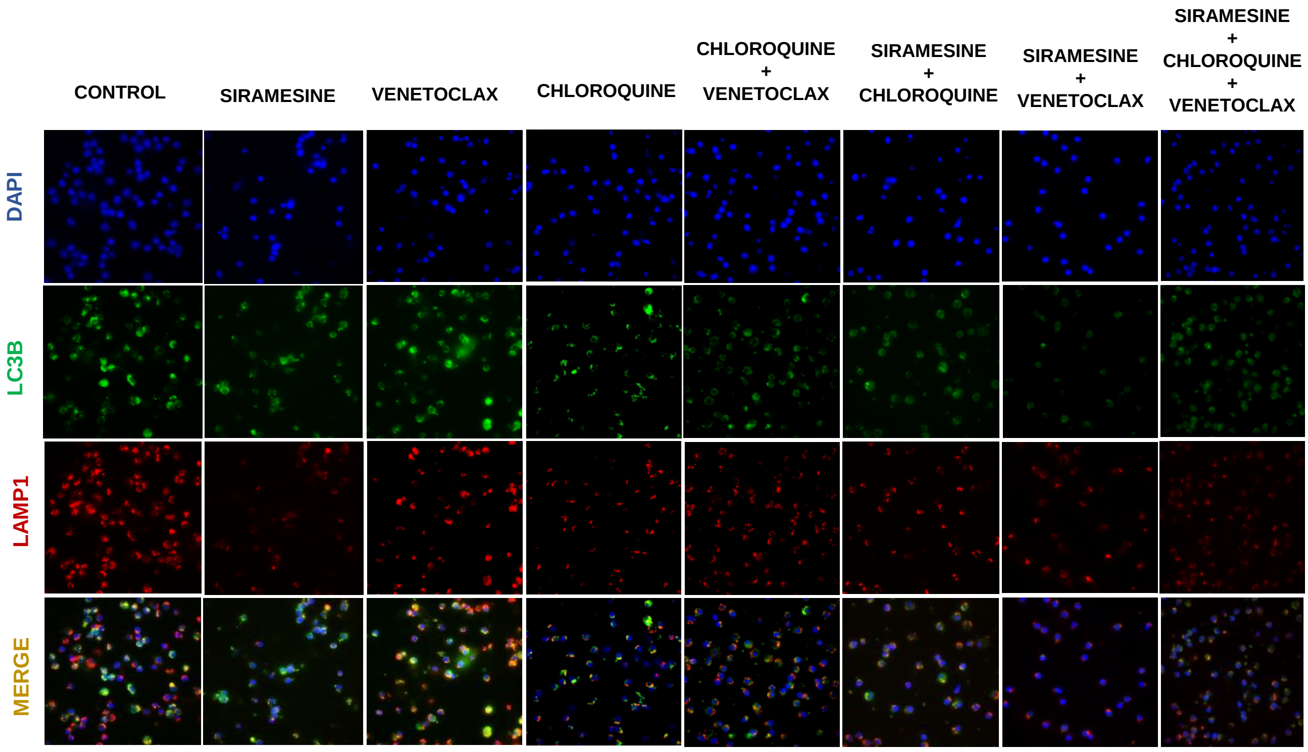

Supplementary Figure S8: Siramesine inhibits autolysosome formation in CLL cells. A) CLL cells were treated with 1µM of siramesine and 0.05nM of venetoclax for 24 hours alone or in the combination with 20µM chloroquine. The representative immunofluorescence staining with antibody against LC3B and LAMP1 is indicated and DAPI stains the nucleus.

Supplementary Figure S9

A)

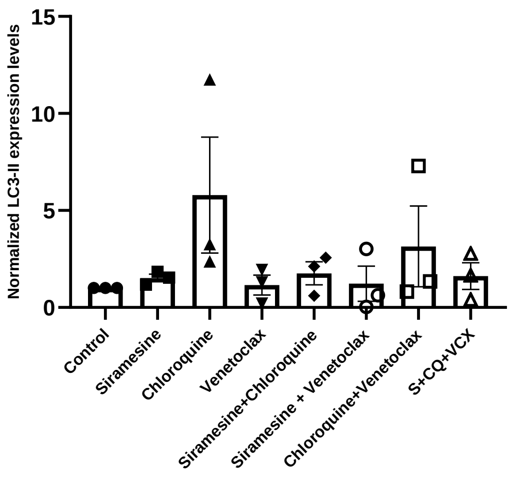

B)

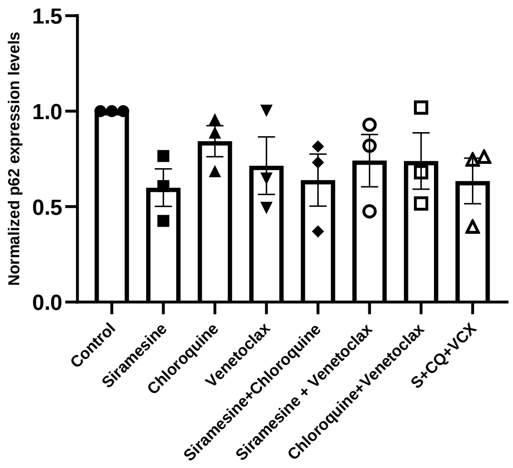

Supplementary Figure S9: Siramesine inhibits autophagy flux in CLL cells. A) CLL cells were treated with 1μM of siramesine, 20 μM of chloroquine and 0.05nM of venetoclax for 24 hours. Autophagy was measured by western blotting for autophagy marker protein LC-II in the absence and presence of lysosomal inhibitor chloroquine. B) SQSTM1/p62, a protein substrate of autophagy was also measured by western blot. Densitometry was performed on three independent different patient samples. Error bars represent standard error of the mean.

Supplementary Figure S10

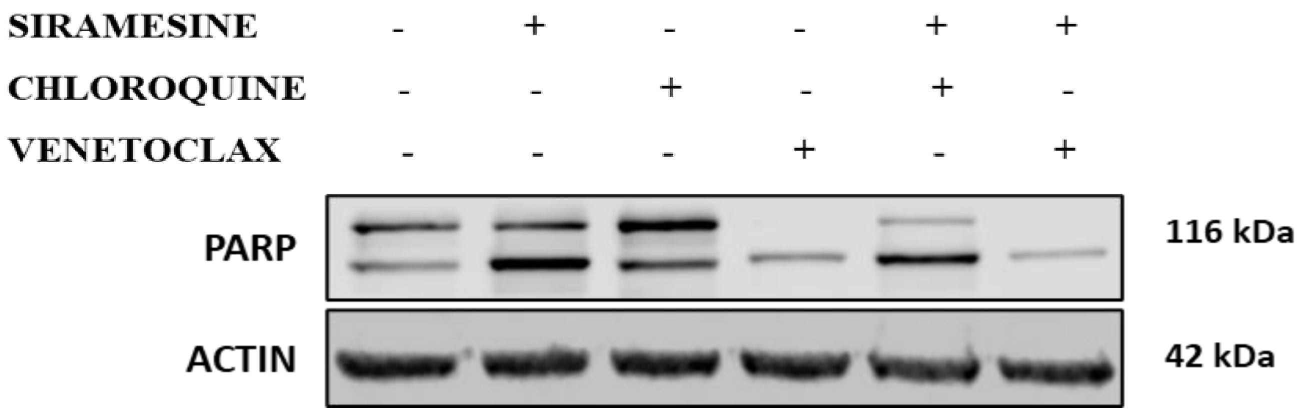

Supplementary figure S10: Induction of apoptosis by the combination of siramesine and venetoclax. CLL cells were treated with 1µM siramesine, 20 µM chloroquine, 0.05nM venetoclax alone and in combination for 24 hours. After treatment, cells were lysed, western blot determination of PARP was performed and actin was used as loading control. Densitometry was calculated (n=3).
